# Bridging Minds and Behaviours: Patient Empowerment Mediates Psychological Factors and Diabetes Self‐Management

**DOI:** 10.1002/edm2.70183

**Published:** 2026-03-03

**Authors:** Shujie Liu, Fei Chen, Fangli Tang, Wenjun Wang, Xiaodan Yuan, Dan Cheng, Yetong Wang, Qingqing Lou

**Affiliations:** ^1^ Hainan Clinical Research Center for Metabolic Disease The First Affiliated Hospital of Hainan Medical University Haikou Hainan China; ^2^ College of Nursing Henan University of Science and Technology Luoyang Henan China; ^3^ Department of Endocrinology The First Affiliated Hospital of Hainan Medical University Haikou Hainan China; ^4^ Department of Public Health, Affiliated Hospital of Integrated Traditional Chinese and Western Medicine Nanjing University of Chinese Medicine Nanjing Jangsu China; ^5^ Department of Hematology Peking University Shenzhen Hospital Shenzhen Guangdong China; ^6^ Department of Renal Department and Hemodialysis The First Affiliated Hospital of Henan University of Science and Technology Luoyang Henan China

**Keywords:** diabetes distress, empowerment, self‐management behaviours, theory of planned behaviour, Type D personality

## Abstract

**Objectives:**

This multicenter cross‐sectional study was designed to verify whether patient empowerment mediates the relationship between psychosocial factors and diabetes self‐management behaviours and HbA1c. Patient empowerment is a key factor improving diabetes management. The mechanism underneath is less clear.

**Methods:**

We conducted a multicenter cross‐sectional study of 2005 type 2 diabetes from five university hospitals across diverse Chinese regions between December 2022 and May 2023. Demographic and lab values came from electronic medical record systems (EMS) while psychological factors were assessed via self‐administered digital scales on a WeChat mini‐program. The study examined patient empowerment's mediating role between psychological factors (Type D personality, DD, attitudes, subjective norms, perceived control, behaviour change intentions) and diabetes outcomes using mediation and chain mediation analyses.

**Results:**

Patients with lower empowerment scores had shorter disease duration, poorer glycemic control, a higher prevalence of dyslipidemia and multiple complications, and greater socioeconomic disadvantage. Type D personality (OR = 2.201, *p* < 0.001) and diabetes distress (DD, OR = 1.053, *p* < 0.001) were identified as independent risk factors for low empowerment. Moreover, patient empowerment significantly mediated the associations between psychosocial factors and both self‐management behaviours and HbA1c. Mediation analyses showed significant indirect effects of Type D personality (*γ* = −0.794, *p* < 0.001), DD (*γ* = −0.050, *p* = 0.024), attitude toward behaviour change (*γ* = 0.470, *p* < 0.001), subjective norm (*γ* = 0.287, *p* < 0.001), perceived behavioural control (*γ* = 0.489, *p* < 0.001) and behavioural intention (*γ* = 0.458, *p* < 0.001) on self‐management behaviours through empowerment.

**Conclusion:**

Patient empowerment functions as a critical mediator linking psychosocial factors to self‐management behaviours and glycemic control in individuals with type 2 diabetes. Type D personality and DD represent important risk factors for low empowerment. These findings suggest that patient empowerment‐focused interventions may enhance diabetes self‐management and glycemic control.

## Graphical abstract

This graphical abstract illustrates the conceptual framework of the study, showing how psychosocial factors influence glycemic control with type 2 diabetes through patient empowerment and self‐management behaviours.

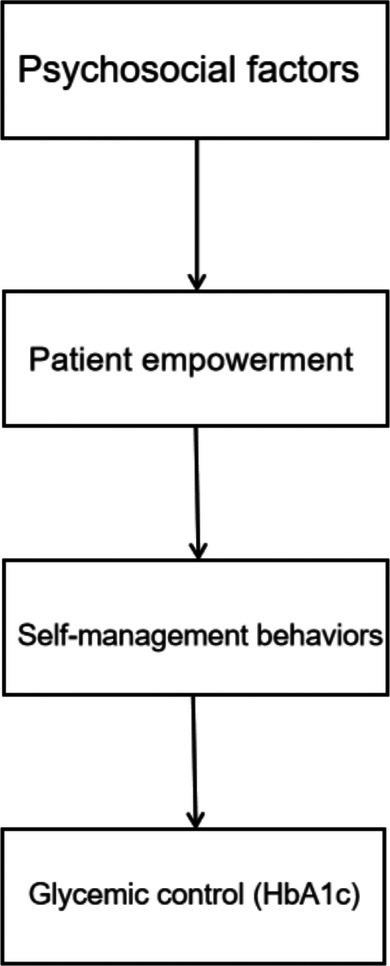

## 1 Introduction

According to the Global Burden of Disease Study 2021 (GBD 2021), diabetes affected an estimated 529 million individuals globally in 2021, representing a striking 6.1% of the world's adult population. Alarmingly, epidemiological modelling projects a 2.5‐fold surge in diabetes prevalence to 1.31 billion cases by 2050, outpacing global population growth rates [1]. Suboptimal glycemic control precipitates debilitating micro‐ and macrovascular complications—notably cardiovascular disease (HR = 2.14, 95% CI [1.89–2.42]), end‐stage renal disease (annual incidence 8.7%) and vision‐threatening retinopathy (prevalence 28.8% in T2DM)—collectively reducing life expectancy by up to 12 years while severely compromising quality‐adjusted life years [2], imposing substantial healthcare expenditures [3].

Diabetes self‐management serves as the cornerstone of diabetes care [4] and plays a pivotal role in disease prognosis. Effective self‐management practices—such as maintaining a healthy diet, engaging in regular physical activity, adhering to prescribed medications and consistent monitoring—can improve glycemic, blood pressure and lipid control in individuals with diabetes, thereby preventing or delaying the onset of complications [5, 6]. Notably, optimal self‐management can reduce Glycated haemoglobin (Haemoglobin A1c, HbA1c) by at least 0.6%, an effect equivalent to the glucose‐lowering efficacy of many antidiabetic medications, without significant adverse effects [4]. Despite nationwide implementation of standardised diabetes education programs since the early 2000s, diabetes self‐management remains suboptimal [7], and population‐level glycemic control rates remain below 40% [8]. Recent multicenter studies reveal only 28.6% of patients achieve optimal self‐monitoring of blood glucose frequency, whereas medication adherence stagnates at 63.2%—significantly lower than Japan's 81.4% and South Korea's 78.9% [8, 9].

A growing body of evidence demonstrates that patient empowerment serves as a potent strategy for improving diabetes self‐management outcomes [10]. Defined as an individual's ability to actively engage in healthcare decision‐making and proactively access support systems during self‐care activities [11], patient empowerment operates through three key mechanisms: strengthening the ability of decision‐making [12], enabling patients to identify intrinsic barriers through self‐reflection, thereby enhancing self‐management capacity [13], equipping patients with essential knowledge and skills to enhance disease management confidence and optimise self‐management practices and improve quality of life [14], building disease management self‐efficacy, and fostering personal health accountability. These combined effects lead to measurable clinical benefits, including better glycemic control, improved glucose variability, and enhanced quality of life metrics in diabetes populations [15]. Importantly, the motivational impact of patient empowerment creates a virtuous cycle—as patients develop greater competence in self‐management skills, they demonstrate increased adherence to treatment protocols [10], which in turn drives more sustainable HbA1c optimization compared to conventional education approaches [16].

Psychological determinants significantly impact self‐management behaviours in diabetes. Among psychological factors, personality traits serve as significant predictors of self‐management behaviours in individuals with type 2 diabetes [17]. Evidence from a meta‐analysis confirms that these traits play a pivotal role in maintaining healthy lifestyles and directly influence self‐management adherence tailored to individual needs among type 2 diabetes patients [18]. Diabetes Distress, defined as the emotional burden arising from the management and treatment of diabetes, was significantly associated with suboptimal glycemic control as well as inadequate self‐management [19]. Behavioural attitudes, subjective norms [20], and Perceived behavioural control [21] constitutes a critical determinant of self‐management behaviours in type 2 diabetes. Furthermore, behavioural intention exhibits a relationship with self‐management behaviours, where greater intention strength predicts more favourable diabetes management outcomes [22].

Research has identified multiple psychosocial determinants—including personality traits, DD, self‐management attitudes, perceived control, and social support—that substantially influence patient empowerment levels. Empirical studies have confirmed that specific personality characteristics (e.g., conscientiousness and openness) show positive correlations with patient empowerment [23], whereas DD demonstrates a strong inverse relationship [24], suggesting that alleviating distress may enhance patient empowerment. Moreover, intervention studies indicate that improving patients' behavioural attitudes, strengthening family/social support systems, and developing volitional control can effectively boost empowerment outcomes in diabetes self‐management [25].

**FIGURE 1:**
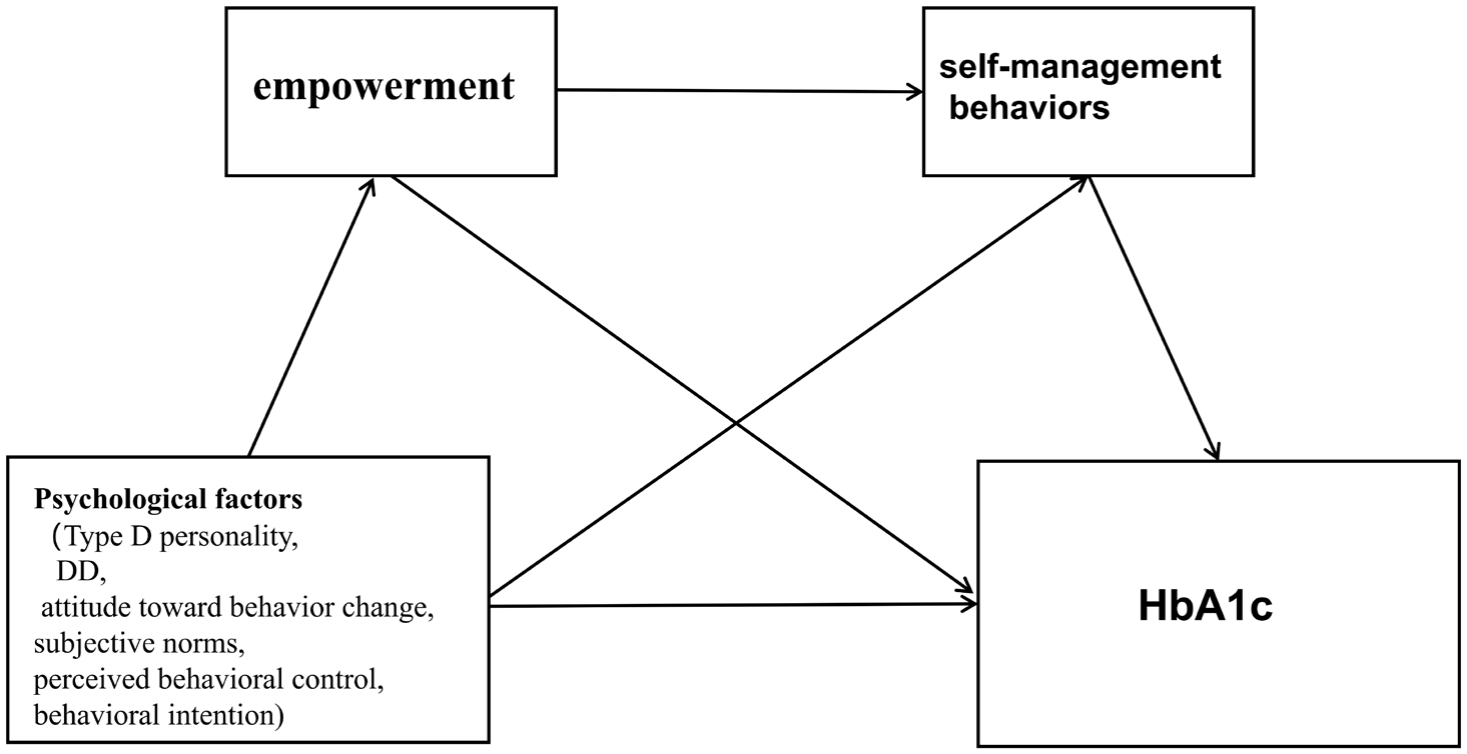
Conceptual model of the proposed mediation pathways. This conceptual diagram illustrates the hypothesised mediation model in which psychological factors (including Type D personality, diabetes distress, attitudes toward behaviour change, subjective norms, perceived behavioural control and behavioural intention) directly and indirectly (through patient empowerment) affect self‐management behaviours and HbA1c. DD, diabetes distress; HbA1c, haemoglobin Alc.

In summary, substantial evidence confirms that patient empowerment serves as a fundamental driver of effective self‐management practices, whereas psychological factors, particularly Type D personality traits, DD, self‐management attitudes, social support, and perceived control, demonstrate significant influence on empowerment levels. However, prior studies rarely explored patient empowerment as a mediator integrating multiple psychosocial determinants in Chinese diabetic populations. Building upon this evidence, we specifically examine whether these psychological variables indirectly impact both self‐management behaviours and HbA1c through their effects on patient empowerment. Furthermore, this study seeks to identify key predictors of low empowerment states, which may represent critical intervention targets for improving diabetes care outcomes. We hypothesised that patient empowerment mediates the relationship between psychological predictors (Type D personality, diabetes distress and TPB components) and both self‐management behaviours and glycemic control. The conceptual model of the proposed mediation pathways is presented in Figure 1.

## 2 Materials and Methods

### 2.1 Study Population

#### 2.1.1 Recruitment and Setting

We conducted a multicenter cross‐sectional analytical study involving consecutive patients recruited from the endocrinology departments of five tertiary grade‐A general university hospitals located in Hainan, Jiangsu, Zhejiang and Henan provinces between December 2022 and May 2023.

Eligible participants who met all of the following criteria were included in this study: (1) Diagnosis of type 2 diabetes mellitus according to the 1999 WHO diagnostic criteria; (2) minimum disease duration of 6 months; (3) age between 18 and 75 years; (4) adequate literacy to complete self‐reported questionnaires; (5) normal cognitive function without communication impairments and (6) willingness to participate in this study and provide informed consent.

**FIGURE 2:**
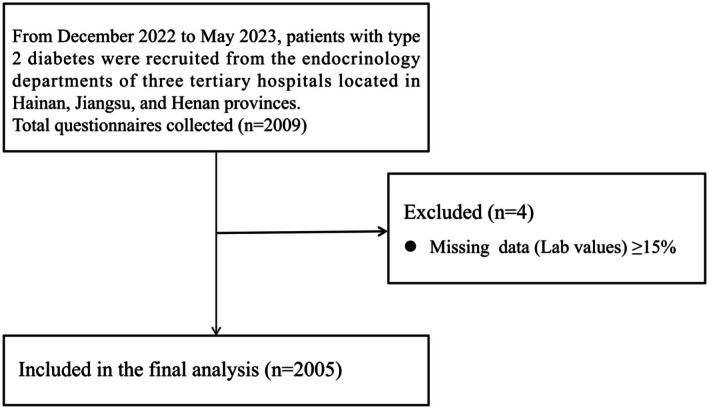
Flowchart of participant selection.

Participants were excluded if they presented with: (1) Severe comorbidities (cardiac, hepatic, renal or cerebrovascular) significantly affecting daily living or self‐management capacity; (2) visual impairments precluding questionnaire completion; (3) physical disabilities requiring assistance for basic activities of daily living or (4) current pregnancy or lactation status. The flowchart of participant selection is presented in Figure 2.

#### 2.1.2 Ethical Considerations

The study protocol received ethical approval from the Institutional Review Board of the First Affiliated Hospital of Hainan Medical University (2022 [Scientific Research] No. 37). All participants provided written informed consent after receiving detailed information about the study procedures, potential risks and benefits.

### 2.2 Data Collection

The study protocol was implemented following formal approval from department leadership. Research personnel conducted systematic screening of potential participants through comprehensive electronic medical record (EMR) reviews to verify eligibility status. A specially trained survey team, consisting of two research associates and two certified diabetes nurse specialists, completed a standardised training program and demonstrated competency through formal assessments before commencing data collection activities. All enrolled participants received detailed explanations regarding the research objectives, methodological procedures, and scientific significance of the study. Psychological factors were assessed through self‐administered digital scales delivered via a dedicated WeChat mini‐program. Participants autonomously completed all psychological measures (including personality traits, DD, behavioural attitude, subjective norms, perceived behavioural control, behavioural intention and self‐management behaviours scales) on their mobile devices. All questionnaire data were completed by participants on‐site and verified in real‐time to ensure 100% completeness. For demographic and laboratory data, multiple imputation methods were applied when the missing rate was less than15%. Cases with a missing rate exceeding 15% were deleted. The program incorporated multiple data quality control features: (1) response progress tracking, (2) logical consistency checks and (3) mandatory completion requirements. Researchers monitored submission status in real‐time through a backend management system, with automated reminders sent for incomplete questionnaires. Relevant demographic characteristics and laboratory indices were systematically extracted from institutional EMR systems.

To reduce selection bias, this study adopted a multicenter design and included data from five hospitals across four provinces: Hainan, Jiangsu, Zhejiang and Henan. Self‐report bias was minimised by administering questionnaires anonymously in a quiet environment. Uniform investigator training was applied to five researchers to perform standardised data collection. Moreover, any data points identified as extreme, anomalous or logically inconsistent were subject to immediate on‐site verification with the corresponding patients. The questionnaires were administered to patients in two distinct phases, thereby minimising common method bias, and Harman single‐factor test was applied exam the common‐method bias.

### 2.3 General Information and Lab Values

The study collected comprehensive demographic, clinical and biochemical data from electronic medical records including age, sex, anthropometric measurements (height, body mass index and waist‐to‐hip ratio), diabetes duration, blood pressure parameters (systolic and diastolic), lifestyle factors (smoking status and alcohol consumption), sociodemographic characteristics (marital status, education level and monthly income), family history of diabetes, diabetes‐related complications and relevant comorbidities such as hypertension and dyslipidemia. Laboratory parameters including HbA1c and complete lipid profiles were also systematically retrieved from the EMS.

### 2.4 Diabetes Empowerment Scale Short Form (DES‐SF)

The DES‐SF, originally developed by Anderson et al. [26], is an 8‐item instrument designed to assess patients' intrinsic capacity for diabetes self‐management. A higher total score on the scale indicates a higher level of patient self‐management potential. Following its initial validation, Hu et al. [27] conducted rigorous translation and cultural adaptation of the scale for Chinese populations. Psychometric evaluation of the Chinese version demonstrated excellent reliability, with a Cronbach's *α* coefficient of 0.848 indicating strong internal consistency and a test–retest reliability of 0.817 confirming measurement stability over time.

### 2.5 Summary of Diabetes Self‐Care Activities (SDSCA)

Diabetes self‐management behaviours were evaluated using the Chinese version of SDSCA measure. The original English version was developed by Toobert et al. [28], with subsequent translation and cultural adaptation conducted by Wan et al. [29] through standardised validation procedures. Psychometric evaluation of the Chinese version revealed adequate internal consistency (Cronbach's *α* = 0.62) and excellent test–retest reliability (*r* = 0.83). Higher scale scores indicate better self‐management behaviours.

### 2.6 Type D Personality Scale (DS‐14)

The study utilised the Chinese version of the DS‐14 scale, originally revised by Denollet [30] and subsequently culturally adapted and validated by Yu and Zhang [31]. This 14‐item instrument consists of two 7‐item subscales measuring negative affectivity (NA) and social inhibition (SI), with items 1 and 3 requiring reverse scoring. Participants were classified as having a Type D personality only when both NA ≥ 10 and SI ≥ 10; otherwise, they were categorised as non‐Type D.

### 2.7 5‐Item Problem Areas in Diabetes Survey (PAID‐5)

The PAID‐5 was utilised to measure diabetes‐related emotional distress in the study. Initially developed and validated by Polonsky et al. [32], the instrument was subsequently cross‐culturally adapted for Chinese populations by Huang et al. [33] through standardised translation and validation procedures. The Chinese version exhibits robust psychometric properties, with excellent internal consistency (Cronbach's *α* = 0.93–0.95) and good temporal stability (test–retest reliability = 0.83). Higher total scores indicate greater levels of diabetes‐related psychological distress.

### 2.8 Behavioural Attitude, Subjective Norms, Perceived Behavioural Control and Behavioural Intention

The assessment of psychological factors of behavioural attitude, subjective norms, perceived behavioural control and behavioural intention was conducted using the Theory of Planned Behaviour‐based Diabetes Self‐Management Predictive Scale (TPB‐DMPS). This instrument was originally developed by Chinese researchers through rigorous application of the Theory of Planned Behaviour framework [34, 35]. Psychometric evaluation established excellent measurement properties, with demonstrated high reliability (Cronbach's *α* = 0.830) and stability (test–retest reliability = 0.950). The TPB‐DMPS comprises 23 items distributed across four theoretical domains: behavioural attitude (6 items), subjective norms (7 items), perceived behavioural control (5 items) and behavioural intention (5 items). Participants responded to all items using a 5‐point Likert scale according to their personal experiences.

The scoring system was implemented as follows: (1) Behavioural attitude items were rated from 1 (‘very unimportant’) to 5 (‘very important’); (2) subjective norm items ranged from 1 (‘very weak’) to 5 (‘very strong’); (3) perceived behavioural control items scaled from 1 (‘very difficult’) to 5 (‘very easy’) and (4) behavioural intention items were scored from 1 (‘very weak’) to 5 (‘very strong’). Higher total scores reflected stronger propensities toward planned diabetes self‐management behaviours.

### 2.9 Statistical Analysis

Statistical analyses were performed using R version 4.2.3 and IBM SPSS Statistics version 26.0. Participants were stratified into low‐empowerment (DES‐SF score < 3) and high‐empowerment (DES‐SF score ≥ 3) groups based on their DES‐S scores. Continuous variables with normal distribution were presented as mean ± standard deviation and analysed using independent *t*‐tests, whereas non‐normally distributed variables were expressed as median (interquartile range, IQR) and compared using Mann–Whitney *U* tests. Categorical variables were reported as frequencies (percentages) and evaluated with chi‐square tests.

Binary logistic regression analysis identified factors associated with low empowerment status. Mediation analyses were conducted to examine the relationships between psychological predictors (Type D personality, DD, attitude toward behaviour change, subjective norms, perceived behavioural control and behavioural intention) and diabetes self‐management behaviours through the mediating role of empowerment. The normality of the mediator and outcome residuals was assessed using Shapiro–Wilk tests and Q‐Q plots. All tests were non‐significant (*p* > 0.05), and the plots showed no substantial deviations. The normality assumption was thus considered met. Multicollinearity among predictors was examined via variance inflation factors (VIFs); all VIF values were well below the conservative threshold of 5, indicating negligible multicollinearity. These analyses were performed in R version 4.2.3, with statistical significance set at *p* < 0.05.

Further serial mediation analysis using the PROCESS macro (version 4.1) in SPSS 26.0 investigated the sequential mediating effects of empowerment and self‐management behaviours on the association between psychological predictors and HbA1c. The bootstrap resampling method with 5000 iterations (Model 6 in PROCESS) was employed to test the significance of these chained mediation pathways (X → M1 → M2 → Y) at *α* = 0.05.

To ensure adequate statistical power for the mediation analyses, a post hoc power analysis was conducted using two complementary approaches: (1) Sobel's analytical approximation, based on the observed path coefficients and their standard errors and (2) a Monte Carlo simulation (5000 replications) assuming normally distributed errors. The Sobel test indicated statistical power of 100%, whereas the Monte Carlo simulation yielded power estimates ranging from 90.3% to 100%. Both methods consistently indicated that our study possessed adequate power to detect the observed indirect effect with a sample size of *N* = 2005. Harman single‐factor test was employed to assess the presence of common method bias.

## 3 Results

### 3.1 Patient Characteristics

The mean empowerment score across all participants was 3.69 ± 0.71 (range: 1–5). Using established cutoff criteria, we classified participants into low empowerment (score < 3, *n* = 411) and high empowerment (score ≥ 3, *n* = 1594) groups. Type D personality was prevalent in 583 patients, accounting for 29.08% of the total sample. Mean scores on psychological measures were as follows: PAID‐5 = 3.76 ± 3.75, TPB = 80.76 ± 11.44, and composite self‐management behaviours = 15.44 ± 6.65. Complete demographic and clinical characteristics, including age, gender, marital status and 19 additional variables, are presented in Table 1.

**TABLE 1:** Patient characteristics.

Variable	Total (*n* = 2005)	Low empowerment group (*n* = 411)	High empowerment group (*n* = 1594)	*t*/*x* ^2^/*Z*	*p*
Age ($\text{years},\bar{x}\pm s$)	56.60 ± 11.22	56.14 ± 10.95	56.72 ± 11.28	−0.930	0.352
Disease duration (years, *M* [Q1, Q2])	7 (2, 13)	5 (1, 10)	8 (2, 13)	−5.215	< 0.001
Male (*n* [%])	1191 (59.40)	239 (58.15)	952 (59.72)	0.335	0.563
BMI (kg/m^2^, $\bar{x}\pm s$)	24.59 ± 3.68	24.49 ± 3.96	24.62 ± 3.61	−0.646	0.518
WHR ([Q1, Q2])	0.93 (0.89, 0.97)	0.93 (0.89, 0.97)	0.94 (0.89, 0.97)	−0.247	0.805
SBP (mmHg, $\bar{x}\pm s$)	129.44 ± 18.55	130.70 ± 19.87	129.11 ± 18.19	1.551	0.121
DBP (mmHg, $\bar{x}\pm s$)	77.69 ± 11.74	78.46 ± 12.93	77.49 ± 11.41	1.494	0.135
HbA1c (%, *M* [Q1, Q2])	8.7 (7.1, 10.8)	9.7 (7.7, 11.6)	8.5 (7.0, 10.5)	−5.893	< 0.001
Marital status (*n* [%])					
Single	58 (2.89)	10 (2.43)	48 (3.01)	0.833	0.841
Married	1871 (93.32)	387 (94.16)	1484 (93.10)		
Divorced	37 (1.85)	6 (1.46)	31 (1.94)		
Widowed	39 (1.95)	8 (1.95)	31 (1.95)		
Family history of diabetes (*n* [%])	472 (42.45)	156 (37.96)	677 (42.47)	2.743	0.098
Living alone (*n* [%])	833 (41.55)	18 (4.28)	80 (5.02)	0.287	0.592
Residence type (*n* [%])					
Urban	1184 (59.05)	198 (48.18)	986 (61.86)	25.295	< 0.001
Rural	821 (40.95)	213 (51.82)	608 (38.14)		
Smoking (*n* [%])	562 (28.03)	128 (31.14)	434 (27.23)	2.484	0.115
Drinking (*n* [%])	324 (16.16)	65 (15.82)	259 (16.25)	0.045	0.831
Education level (*n* [%])					
High school or above	30 (1.50)	3 (0.73)	27 (1.69)	19.214	< 0.001
Secondary school	1129 (56.31)	270 (65.69)	859 (53.89)		
Primary school or below	846 (42.19)	138 (33.58)	708 (44.42)		
Comorbidities (*n* [%])					
Hypertension	834 (41.59)	156 (37.96)	678 (42.53)	2.819	0.093
Dyslipidemia	856 (42.69)	207 (50.37)	649 (40.72)	12.436	< 0.001
Number of complications (*n* [%])					
0	953 (47.53)	150 (36.50)	803 (50.38)	25.242	< 0.001
1	666 (33.22)	95 (23.11)	337 (21.14)		
2	212 (10.57)	104 (25.30)	281 (17.63)		
≥ 3	174 (8.68)	62 (15.09)	173 (10.85)		
Monthly income (*n* [%])					
< 1000	214 (10.67)	72 (17.52)	142 (8.91)	48.702	< 0.001
1000–2000	225 (11.22)	62 (15.09)	163 (10.22)		
2000–3000	373 (18.60)	89 (21.65)	284 (17.82)		
3000–5000	513 (25.59)	83 (20.19)	430 (26.98)		
> 5000	680 (33.92)	105 (25.55)	575 (36.07)		
Health insurance (*n* [%])					
Employment‐based insurance (85%)	904 (45.09)	121 (29.44)	783 (49.12)	71.648	< 0.001
Resident basic medical insurance (55%)	1010 (50.37)	276 (67.15)	734 (46.05)		
Self‐funded medical care (0%)	91 (4.54)	14 (3.41)	77 (4.83)		

*Note:* No missing data for any variable. Data are presented as mean ± standard deviation (SD) for normally distributed continuous variables, median (interquartile range, IQR) for non‐normally distributed continuous variables or *n* (%) for categorical variables.

Abbreviations: BMI, body mass index; DBP, diastolic blood pressure; HbA1c, haemoglobin Alc; SBP, systolic blood pressure; WHR, waist‐to‐hip ratio.

### 3.2 Factors Associated With Low Empowerment

**FIGURE 3:**
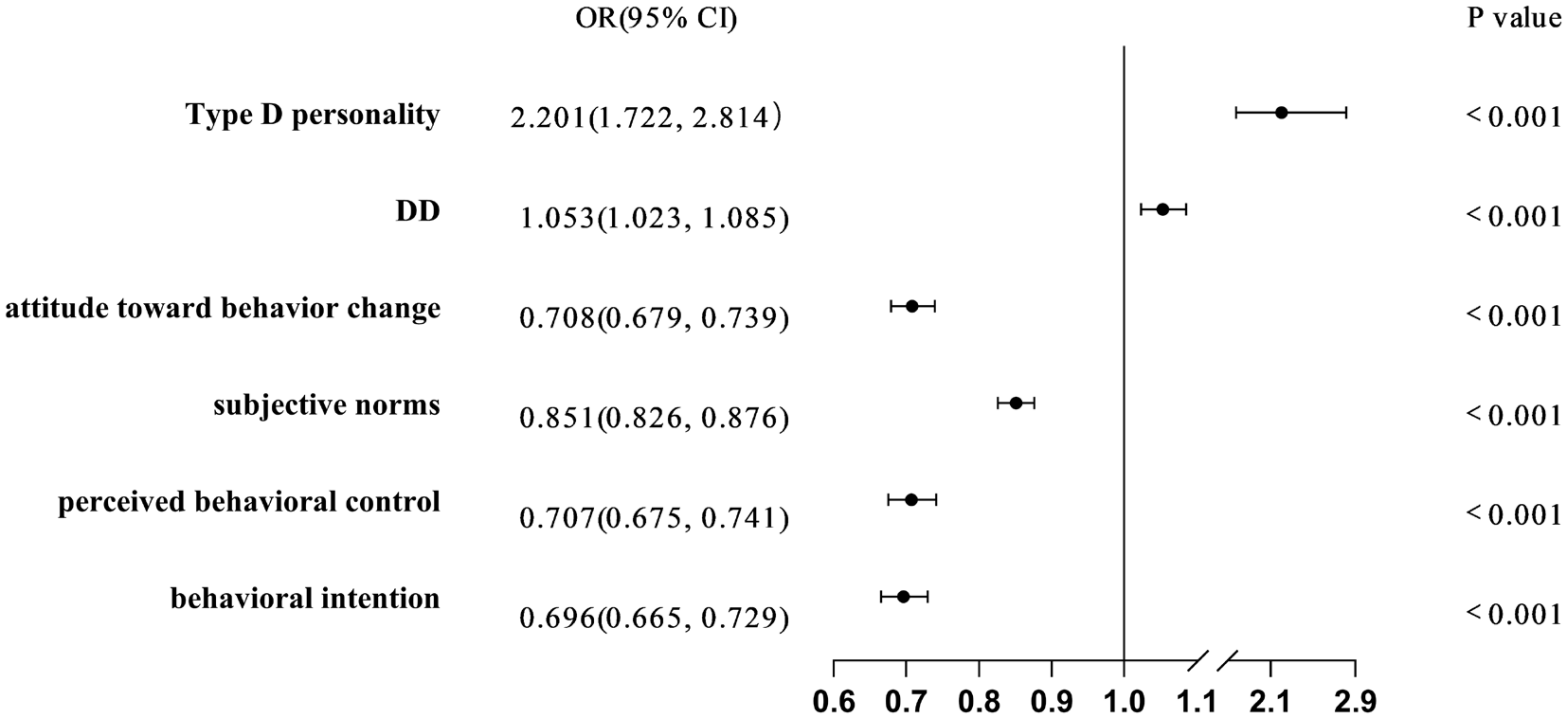
Risk factors of low empowerment. Risk factors for low empowerment were analysed using odds ratios (OR) with 95% confidence intervals (CI), adjusting for age, sex, disease duration, monthly income, education level, marital status, living alone, residence type and family history of diabetes. OR > 1 indicates a risk factor for low empowerment, whereas an OR < 1 indicates a protective factor against low empowerment. DD, diabetes distress.

Binary logistic regression identified significant predictors of low empowerment. Type D personality (OR = 2.201, 95% CI [1.722, 2.814], *p* < 0.001) and DD (OR = 1.053, 95% CI [1.023, 1.085], *p* < 0.001) were independently associated with increased risk of low empowerment. Thus, Type D personality and DD constitute significant risk factors for low empowerment. In contrast, behavioural attitudes (OR = 0.708, 95% CI [0.679, 0.739], *p* < 0.001), subjective norms (OR = 0.851, 95% CI [0.826, 0.876], *p* < 0.001), perceived behavioural control (OR = 0.707, 95% CI [0.675, 0.741], *p* < 0.001) and behavioural intention (OR = 0.696, 95% CI [0.665, 0.729], *p* < 0.001) were positively associated with patient empowerment. Full results are presented in Figure 3.

### 3.3 Mediation Analyses Revealed Significant Indirect Effects of Empowerment Between Psychological Factors and Self‐Management Behaviours

The mediation model examining empowerment as a mediator revealed significant total effects of Type D personality (*γ* = 0.976, *p* = 0.003), DD (*γ* = 0.079, *p* = 0.046), attitude toward behaviour change (*γ* = 0.705, *p* < 0.001), subjective norms (*γ* = 0.552, *p* < 0.001), perceived behavioural control (*γ* = 1.088, *p* < 0.001) and behavioural intention (*γ* = 1.116, *p* < 0.001) on self‐management behaviours. Significant indirect effects through empowerment were observed for all predictors including Type D personality (*γ* = −0.794, 95% CI [−1.219, −0.384]), DD (*γ* = −0.050, 95% CI [−0.004, −0.097]), attitude toward behaviour change (*γ* = 0.470, 95% CI [0.404, 0.542]), subjective norms (*γ* = 0.287, 95% CI [0.243, 0.333]), perceived behavioural control (*γ* = 0.489, 95% CI [0.421, 0.564]) and behavioural intention (*γ* = 0.458, 95% CI [0.393, 0.522]). For other predictors, the persistence of significant direct effects alongside indirect effects indicated partial mediation. Complete statistical results are presented in Table 2.

**TABLE 2:** Mediating effects of empowerment between psychosocial factors and self‐management behaviours in type 2 diabetes.

	Total effect				Direct effect				Indirect effect			
	*γ*	Lower	Upper	*p*	*γ*	Lower	Upper	*p*	*γ*	Lower	Upper	*p*
Type D personality	0.976	0.335	1.616	0.003	1.769	1.246	2.292	< 0.001	−0.794	−1.219	−0.384	< 0.001
DD	0.079	0.001	0.156	0.046	0.129	0.066	0.193	< 0.001	−0.050	−0.004	−0.097	0.024
Attitude toward behaviour change	0.705	0.631	0.779	< 0.001	0.235	0.160	0.311	< 0.001	0.470	0.404	0.542	< 0.001
Subjective norms	0.552	0.492	0.611	< 0.001	0.265	0.209	0.321	< 0.001	0.287	0.243	0.333	< 0.001
Perceived behavioural control	1.088	1.001	1.175	< 0.001	0.598	0.509	0.688	< 0.001	0.489	0.421	0.564	< 0.001
Behavioural intention	1.116	1.035	1.196	< 0.001	0.658	0.573	0.743	< 0.001	0.458	0.393	0.522	< 0.001

*Note:* Total effect: overall association between psychosocial factors and self‐management. Direct effect: association after accounting for patient empowerment. Indirect effect: mediation by empowerment; significant values indicate partial or full mediation. *γ* > 0: positive, *γ* < 0: negative. *p* < 0.05 denotes statistical significance.

Abbreviation: DD, diabetes distress.

### 3.4 Serial Mediation Analysis Among Psychosocial Factors, Self‐Management Behaviours and HbA1c in Type 2 Diabetes

**FIGURE 4:**
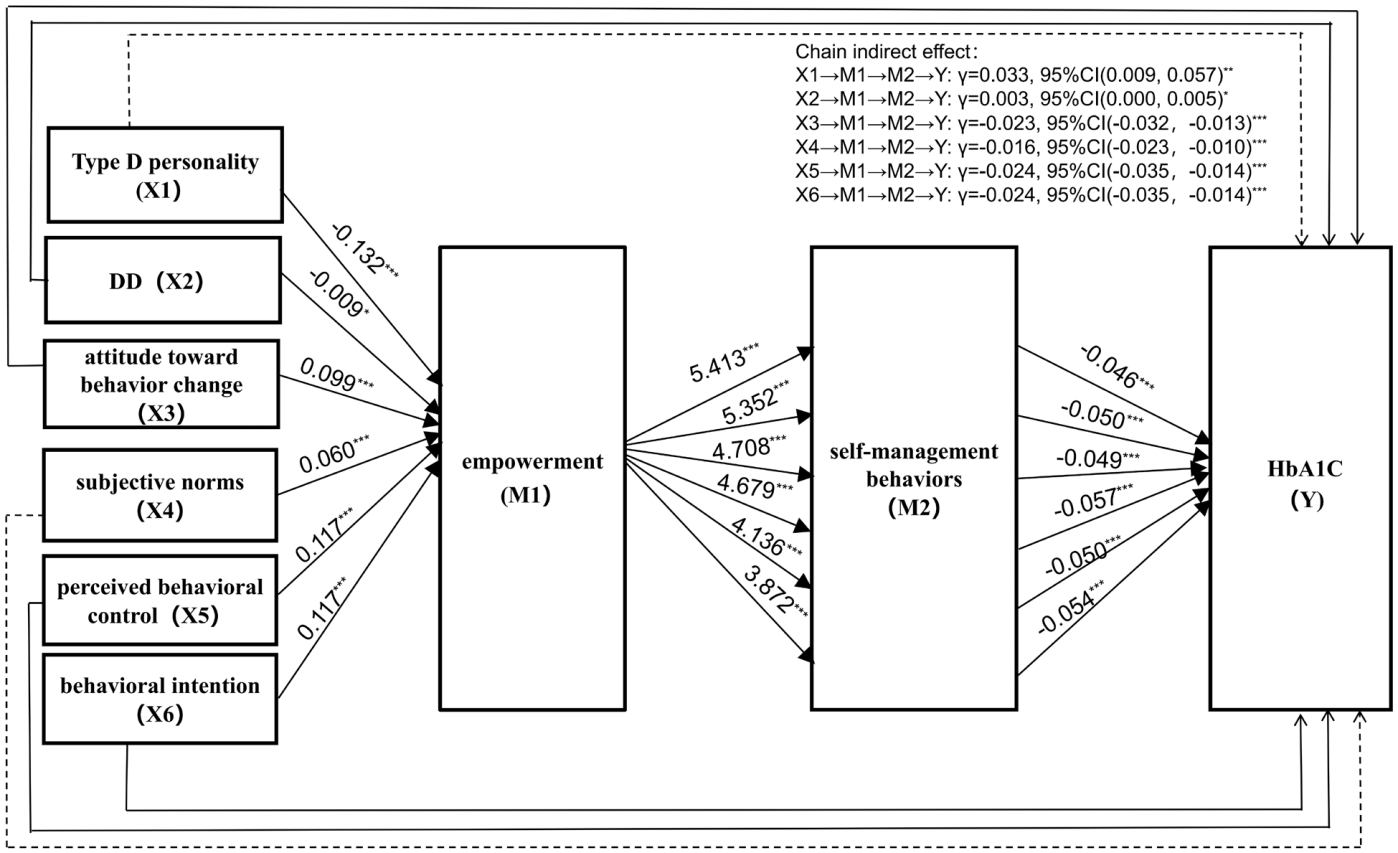
The chain mediating effects between Type D personality, DD, attitude toward behaviour change, subjective norms, perceived control, behavioural intention, empowerment, self‐management behaviours and HbA1c in patients with type 2 diabetes. Figure 4 presents the results of a serial mediation model, illustrating the indirect effects of six independent variables—Type D personality (X1), DD (X2), attitude toward behaviour change (X3), subjective norm (X4), perceived behavioural control (X5) and behavioural intention (X6)—on the outcome variable, HbA1c (Y), via two mediators: empowerment (M1) and self‐management behaviours (M2). Solid lines represent statistically significant mediation pathways, whereas dashed lines indicate non‐significant pathways. Path coefficients (*γ*) and their 95% confidence intervals are reported in the figure. DD, diabetes distress. *p < 0.05; **p < 0.01; ***p < 0.001.

This study employed serial mediation analysis (PROCESS macro Model 6) to systematically examine the hypothesised pathways connecting psychological factors (Type D personality, DD and theory of planned behaviour components including attitude, subjective norms, perceived behavioural control and behavioural intention) to HbA1c through the sequential mediators of patient empowerment (M1) and self‐management behaviours (M2), with the complete mediation model (X → M1 → M2 → Y) illustrated in Figure 4.

The path analysis revealed significant predictive relationships across all measured constructs. Type D personality (*γ* = −0.132, *p* < 0.001), DD (*γ* = −0.009, *p* < 0.05) and attitude toward behaviour change (*γ* = 0.099, *p* < 0.001) each showed negative associations with empowerment. Conversely, subjective norms (*γ* = 0.060, *p* < 0.001), perceived behavioural control (*γ* = 0.117, *p* < 0.001) and behavioural intention (*γ* = 0.117, *p* < 0.001) demonstrated positive predictive effects on empowerment. Notably, self‐management behaviours consistently exhibited significant negative effects on HbA1c levels across all pathways (range: *γ* = −0.046 to −0.057, all *p* < 0.001), indicating improved glycemic control with better self‐management practices.

The results further demonstrated significant indirect effects of psychological factors on HbA1c through the empowerment‐self‐management pathway. Notably, Type D personality showed a positive indirect effect (*γ* = 0.033, 95% CI [0.012, 0.060], *p* < 0.05), whereas DD exhibited a marginal yet significant positive effect (*γ* = 0.003, 95% CI [0.000, 0.005], *p* < 0.05). Conversely, theory of planned behaviour components revealed consistent negative indirect effects on HbA1c: attitude toward behaviour change (*γ* = −0.023, 95% CI [−0.032, −0.013]), subjective norms (*γ* = −0.016, 95% CI [−0.023, −0.010]), perceived behavioural control (*γ* = −0.024, 95% CI [−0.035, −0.014]) and behavioural intention (*γ* = −0.024, 95% CI [−0.035, −0.014]), all showing significant mediation effects (*p* < 0.001) through the sequential pathway of empowerment and subsequent self‐management behaviours. These findings collectively suggest that while Type D personality and DD may have counterintuitive positive associations with glycemic control, standard psychological constructs demonstrate the expected beneficial effects through the hypothesised mediation mechanism.

## 4 Discussion

The study identified patient empowerment as a crucial mediator in the relationship between psychological factors and both diabetes self‐management behaviours and HbA1c. The examined psychological determinants encompassed stable personality characteristics (Type D personality), DD and cognitive‐behavioural components from the theory of planned behaviour (attitudes toward self‐management, subjective norms and perceived behavioural control). These psychological factors demonstrated dual pathways of influence: they exerted both direct effects on self‐management outcomes and glycemic control, as well as indirect effects through the mediating mechanism of patient empowerment. Overall, the findings highlight the important role of patient empowerment in linking psychological characteristics with diabetes management and metabolic outcomes.

Our findings indicate that Type D personality is significantly associated with reduced patient empowerment. Previous research has demonstrated that individuals with Type D personality typically experience elevated stress levels [36]. In the context of type 2 diabetes, chronic and unmanaged stress has been associated with dysregulation of the hypothalamic–pituitary–adrenal axis and increased secretion of stress hormones like cortisol, which may be related to reduced patient empowerment. Patients exhibiting Type D personality characteristics demonstrate distinct behavioural patterns that significantly impact diabetes management. These individuals typically display problem‐avoidance tendencies and passive self‐management behaviours, which are associated with substantially lower levels of empowerment [37]. This evidence underscores the clinical importance of implementing targeted interventions that focus on building self‐confidence and fostering active engagement in health management processes for this patient population.

This study confirmed a significant negative association between DD and patient empowerment, a finding consistent with prior research [24]. Empirical evidence from an Indian study [38] elucidates the underlying psychosocial mechanisms, demonstrating how DD erodes empowerment through the development of maladaptive cognitive patterns. Specifically, affected patients frequently adopt fatalistic beliefs marked by anticipatory failure expectations, which are linked to diminished perceived control and self‐efficacy in diabetes self‐management. The robust inverse relationship between DD and empowerment observed in our study carries important clinical implications. Elevated diabetes‐related psychological distress has been consistently associated with lower empowerment and poorer adherence to self‐care behaviours. These findings highlight the critical need for comprehensive interventions that address psychological adaptation to illness, empowerment enhancement strategies and coping skill development for diabetes‐related psychosocial challenges.

Our study demonstrates a significant positive association between patient empowerment and diabetes self‐management behaviours. Our findings indicate that higher empowerment levels are associated with better self‐care practices and more favourable HbA1c levels, which is consistent with established literature [39]. The observed relationship can be explained through empowerment's dual mechanism of action: it fosters critical health decision‐making capabilities while simultaneously promoting personal responsibility for disease management [12]. These results are noteworthy, as they align with prior clinical evidence indicating that patient empowerment‐based diabetes education interventions are associated with improvements in HbA1c levels [16]. The consistency between our findings and existing research underscores the importance of patient empowerment as a key determinant of successful diabetes self‐management and metabolic outcomes.

Patient empowerment is a multidimensional construct encompassing perceived control, self‐efficacy, active participation in health‐related decision‐making, and behavioural proactivity, and its central role in chronic disease behaviour management has been widely validated [40]. Specifically, empowerment enhances patients' self‐efficacy and sense of control over their illness [41], thereby being associated with the translation of positive health‐related cognitions and external support into concrete self‐management behaviours, such as regular blood glucose monitoring, dietary regulation, and sustained physical activity. In addition, empowerment increases patients' active involvement in health‐related decision‐making and improves the quality of communication with healthcare providers, processes that have been shown to promote treatment adherence and long‐term disease management capacity [12, 13]. Overall, empowerment constitutes an important mediating pathway between psychological factors and diabetes self‐management behaviours as well as metabolic control, allowing multidimensional psychosocial resources to be effectively translated into sustained behavioural engagement and, ultimately, improved glycemic outcomes.

It is possible that there might be reverse causality between patient empowerment and behavioural outcomes. Effective self‐management can lead to multiple positive outcomes for patients, such as reductions in HbA1c levels and a lower incidence of complications, thereby enhancing patients' confidence and further promoting improvements in their empowerment. In addition, the findings of this study may be influenced by cultural factors. Variations in digital literacy among Chinese patients, as well as specific health beliefs and values such as disease perceptions, understanding of self‐management responsibilities, and attitudes toward patient‐physician relationships may affect empowerment perceptions and self‐management behaviours [42].

The enhancement of patient empowerment can be achieved through a multifaceted approach that targets cognitive, social and behavioural dimensions. Specifically, interventions should focus on modifying maladaptive beliefs about self‐management, fortifying familial and social support systems, and augmenting perceived disease control capabilities [43]. These strategies are associated with more constructive illness perceptions and higher self‐efficacy. Consequently, clinical practice should prioritise the cultivation of adaptive health beliefs while actively engaging family networks to provide sustained encouragement, thereby fostering greater confidence in disease management capabilities.

This study offers several methodological and conceptual advances over previous research in this field. Unlike prior investigations that predominantly examined direct effects of isolated psychological factors on self‐management behaviours and HbA1c, our study innovatively identifies patient empowerment as a crucial mediating mechanism through which multiple psychological determinants collectively influence these outcomes. The comprehensive assessment framework, incorporating validated measures including the Type D Personality Scale (DS14), PAID‐5 Scale, and Theory of Planned Behaviour‐based Diabetes Self‐Management Scale, enables multidimensional analysis of both direct and mediated pathways linking psychological factors to behavioural and physiological outcomes. Furthermore, the study's robust epidemiological design, featuring a large, multicenter sample drawn from diverse clinical settings across China, enhances the generalizability of findings and ensures reliable representation of the broader type 2 diabetes population in real‐world clinical practice.

This study has several important limitations that should be considered when interpreting the findings. First, the exclusive focus on hospitalised type 2 diabetes patients may limit the generalizability of results to community‐dwelling populations with potentially different disease characteristics and management approaches. Second, while we examined key psychological factors, residual confounding from unmeasured variables (e.g., socioeconomic status, healthcare access or comorbidities) could influence the observed relationships between personality traits, DD, self‐management attitudes and patient empowerment. Third, questionnaires were self‐reported, which may introduce information bias. Fourth, All questionnaires were collected using the same method. Although we applied program control to minimise common‐method bias, and Harman's single‐factor test indicated that the most important factor explained only 22.14% of the variance, which was below the threshold of 40% [44], there was a chance that common method bias was introduced. Finally, due to the cross‐sectional design, neither temporal nor causal relationships between psychological factors and self‐care behaviours can be inferred. Future longitudinal or intervention studies to test mediation and causality over time are warranted.

In conclusion, psychological factors exert significant effects on patient empowerment, which in turn serves as a critical mediator influencing diabetes self‐management behaviours and glycemic control. These results establish patient empowerment as a pivotal mechanism through which psychological determinants ultimately affect disease management outcomes in type 2 diabetes. The identification of patient empowerment as this key mediating variable offers novel insights into the complex psychosocial pathways underlying self‐management behaviours. From a clinical implementation perspective, these findings strongly advocate for healthcare providers to systematically assess and address psychological barriers to patient empowerment, and incorporate empowerment‐enhancing strategies into standard diabetes education. Integrating empowerment‐based education and psychological assessment into routine diabetes care could enhance self‐management efficacy and improve glycemic outcomes.

## Author Contributions

Shujie Liu: conception and design, statistical analysis and manuscript drafting (equal). Fei Chen: Conception and design, statistical analysis and manuscript drafting (equal). Fangli Tang, Wenjun Wang, Xiaodan Yuan, Dan Cheng and Yetong Wang: data collection and organisation (equal). Qingqing Lou: research guidance, manuscript revision and funding support (equal).

## Funding

This study was supported by the National Natural Science Foundation of China (72164010 and 12561049).

## Ethics Statement

The study protocol received ethical approval from both the Institutional Review Board of the First Affiliated Hospital of Hainan Medical University.

## Conflicts of Interest

The authors declare no conflicts of interest.

## Data Availability

The datasets generated or analysed during the current study are not available publicly because they are subject to national data protection laws and restrictions imposed by the ethics committee to ensure privacy of study participants. However, they can be requested after publication through an individual project agreement with the corresponding author.
